# Impact of SOX2 function and regulation on therapy resistance in bladder cancer

**DOI:** 10.3389/fonc.2022.1020675

**Published:** 2022-11-16

**Authors:** Guodong Chen, Yan Chen, Ruiquan Xu, Guoxi Zhang, Xiaofeng Zou, Gengqing Wu

**Affiliations:** ^1^ The First Clinical College, Gannan Medical University, Ganzhou, China; ^2^ Department of Urology, Affiliated Hospital of Gannan Medical University, Ganzhou, China; ^3^ Institute of Urology, Affiliated Hospital of Gannan Medical University, Ganzhou, China; ^4^ Department of Gastroenterology, Affiliated Hangzhou First People’s Hospital, Zhejiang University School of Medicine, Hangzhou, China

**Keywords:** bladder cancer, SOX2, bladder cancer stem cells, chemoresistant, non-coding RNAs

## Abstract

Bladder cancer (BC) is a malignant disease with high rates of recurrence and mortality. It is mainly classified as non-muscle-invasive BC and muscle-invasive BC (MIBC). Often, MIBC is chemoresistant, which, according to cancer stem cells (CSCs) theory, is linked to the presence of bladder cancer stem cells (BCSCs). Sex-determining region Y- (SRY) Box transcription factor 2 (*SOX2*), which is a molecular marker of BCSCs, is aberrantly over-expressed in chemoresistant BC cell lines. It is one of the standalone prognostic factors for BC, and it has an inherently significant function in the emergence and progression of the disease. This review first summarizes the role of SRY-related high-mobility group protein Box (SOX) family genes in BC, focusing on the *SOX2* and its significance in BC. Second, it discusses the mechanisms relevant to the regulation of *SOX2*. Finally, it summarizes the signaling pathways related to *SOX2* in BC, suggests current issues to be addressed, and proposes potential directions for future research to provide new insights for the treatment of BC.

## 1 Introduction

According to global data on new cases and deaths for all cancers combined in 2020, bladder cancer (BC) ranked 12th in the number of new cases of all cancers, with 573,278 new cases (1). Smoking and long-term exposure to chemical raw materials are two recognized pathogenic factors (2, 3). Bladder cancer is a highly heterogeneous malignant disease that is mainly classified as non-muscle-invasive BC (NMIBC; stages Ta, T1) and muscle-invasive BC (MIBC; stages ≥ T2) (4). Approximately 70% of BC cases diagnosed as NMIBC have a low risk of progression to MIBC, but NMIBC is prone to recurrence. Conversely, uroepithelial flat lesions and carcinoma *in situ* are very vulnerable to progression to MIBC (5). Although some advanced DNA methylation-based urine tests can diagnose BC early and give early treatment, the outcome of BC is still unsatisfactory (6, 7). The main treatments for BC include surgery, chemotherapy and immunotherapy (8). Transurethral resection of bladder tumor combined with bladder perfusion is the main treatment for NMIBC patients (9). Neoadjuvant chemotherapy combined with radical total cystectomy is the standard of treatment for patients with MIBC (10). However, cisplatin-based neoadjuvant chemotherapy (NAC) often causes drug resistance in MIBC patients (11). The use of immune checkpoint inhibitors approved by the US Food and Drug Administration has been unsatisfactory due to low response rates (12). Recent studies report key genes and regulatory pathways in lymphatic metastasis of BC that enhances the anti-tumor effect of cisplatin or immunotherapy (13, 14). Therefore, the development of targeted drugs may provide new clinical strategies to overcome BC chemoresistance.

**Figure 1:**
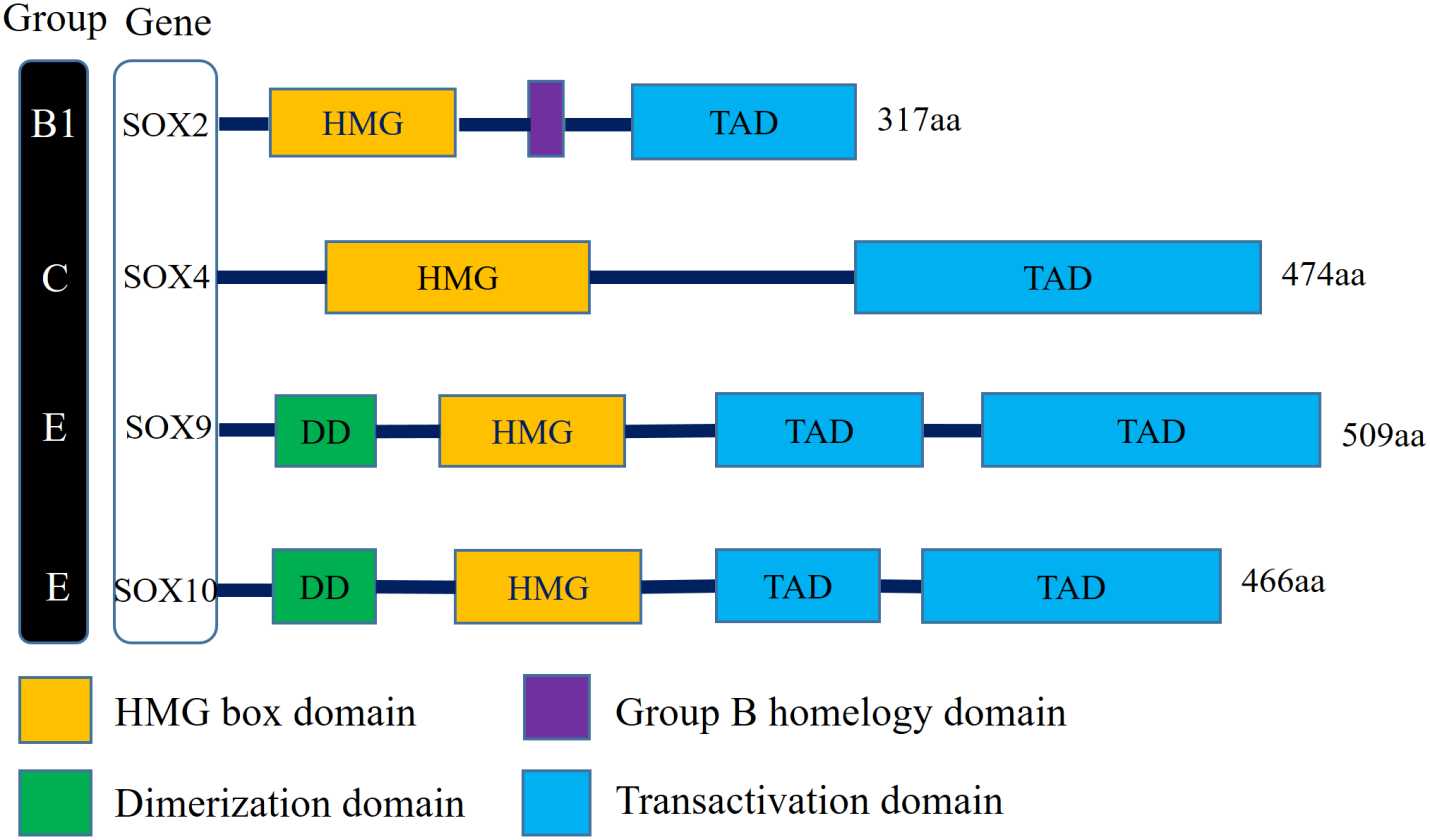
Schematic illustration of structures of the Sox gene family involved in human bladder cancer. SOX2 belongs to SOX family group B1, SOX4 belongs to SOX family group C, SOX9 and SOX10 belong to SOX family group E. All family members have the DNA-binding HMG domain in common. (HMG, High-mobility group box domain; TAD, Transactivation domain; DD, Dimerization domain).

There is a clear correlation between the aberrant expression of sex-determining region Y (SRY) associated high-mobility group (HMG) Box (SOX) transcription factors and carcinomas (15). Numerous studies have demonstrated that several members of the SOX family act as key regulators of tumor cells, which typically mediate the initiation of neoplasms and enhance tumorigenesis and proliferation (16). The SOX family transcription factors modulate tumor cell proliferation, metastasis, stemness, epithelial–mesenchymal transition (EMT), and drug resistance *via* multiple signaling pathways (17). Sex-determining region Y-Box transcription factor 2 (*SOX2*)—a member of the *SOXB1* subgroup—has an encoded product comprising 317 amino peptides. The *SOX2* gene, which is located on chromosome 3 at locus q26.3-q27, is a single exon and intronless gene with a core structure of a highly conserved HMG structural domain that intercalates with specific DNA sequences (18). In addition, *SOX2* and its family members all possess C-terminal trans-activating structural domains, which act to recognize and bind to the promoter regions of target genes, thereby activating or repressing gene expression (19) (**Figure 1**).

This article outlines the link between SOX family members and BC, highlights the *SOX2* as a topic of discussion, and summarizes the mechanisms and signaling pathways that mediate *SOX2* expression to provide new concepts for the management of BC.

## 2 Overview of the human SOX protein family in bladder cancer

The proteins of the SOX family are involved in embryonic development processes, such as cell differentiation and organ formation, and are linked to the maintenance of stem cells in adult tissues (20). In healthy organisms, gene expression is regulated precisely. However, in tumors, *SOX* genes are often dysregulated at the transcriptional, translational, and post-translational levels, and the aberrant activation of *SOX* genes is an important factor in tumorigenesis and progression (17). The SOX family is divided into 8 groups (A–H), with a total of more than 20 family members. In different tumor environments, *SOX* genes can function as oncogenes or tumor suppressor genes. For example, *SOX10* is upregulated in BC and breast cancer and downregulated in colorectal cancer (21). In most tumors, *SOX2*, *SOX4*, *SOX5*, *SOX8*, and *SOX9* act as oncogenes. In BC, the expression of the *SOX2*, *SOX4*, and *SOX10* is upregulated, while the expression of the *SOX9* is downregulated (22) (**Figure 1**).

The *SOX4* gene is a member of the SOXC subgroup, and its expression is upregulated in BC (23). Studies have reported *SOX4* upregulation to be significantly correlated with the grade, invasiveness, and poor outcome of BC (24). *SOX4* is expressed in BC cell lines RT-114, J82, and 5637. Knocking down the expression of *SOX4* can significantly inhibit the proliferation, metastasis, and stemness of BC cells (24, 25). The expression of the *SOX10* gene—a member of the SOXE subgroup—is also upregulated in BC, and the aberrant overexpression of the SOX10 protein is an independent predictive factor for overall survival in BC. In the T24 and 5637 BC cell lines, the expression of *SOX10* can promote the proliferation, migration, and invasion of BC cells (26). The expression of the *SOX9* gene is upregulated in the majority of carcinomas, whereas in BC, *SOX9* expression is inhibited. It has been reported that *SOX9* promoter methylation is upregulated in BC and is significantly associated with BC grade and overall survival. The DNA methyltransferase inhibitor (DNMTi) 5-azacytidine (AZA) treated the BC cell line, and the J82 methylated cell line restored the expression of *SOX9* at the transcriptional level compared with the RT4 unmethylated cell line (27). Finally, a single-cell sequencing analysis reported that *SOX9* expression was higher in muscle-invasive tumors compared to non-muscle-invasive tumors (28). This section summarizes the essential roles of three members of the SOX family in the development and metastasis of BC by describing studies on *SOX4*, *SOX10*, and *SOX9* in BC. Subsequently, we will focus our discussion on *SOX2*.

## 3 Association between *SOX2* and cancer stem cells formation in bladder cancer

With the continued use of drugs, some resistant cell populations selectively survive in tumors, leading to the recurrence of carcinoma, and cancer stem cells (CSCs) are considered as significant contributors to this process (29). Cancer stem cells are a class of undifferentiated cell populations with stem cell characteristics that, in a hierarchical model, sit on the apex of tumor cells; furthermore, they can generate heterogeneous tumor cells and form tumors (30). The characteristics of BCSCs include slow growth and a tendency for dormancy. For slowly dividing cells, chemotherapy based on the DNA damage mechanism has little effect (31). Therefore, exploring key molecules representing the properties of BCSCs is particularly important.

In recent years, CSCs have been demonstrated in and isolated from various solid tumors, such as breast cancer (31). In 2008, She et al. (32) first identified side population (SP) cells (a subset of CSC-enriched subpopulations with the ability to divide asymmetrically, self-renew, and regulate tumor initiation in BC) using DyeCycle Violet reagent (DCV) staining. Subsequently, Yang et al. (33) separated a subset of surface markers CD44v6+/EMA− cells with enhanced capabilities of colony formation, self-renewal, and proliferation. In 2009, Chan et al. (34) isolated a CD44+/CK5+/CK20− cell subset in MIBC that was more tumorigenic in xenograft tumors compared to the CD44-/CK5-/CK20+ subset. In 2013, Peng et al. (35) identified BCSCs in the J82 cell line based on the CD133 cell surface marker. In contrast with the CD133− cell population, the CD133+ cell population was more invasive, tumorigenic, and radiotherapy/chemotherapy resistant. Furthermore, the expression of embryonic stem (ES) cell-related genes *SOX2* and Octamer-binding transcription factor 4 (OCT*4*) was abnormally upregulated in the CD133+ population (35).

In poorly differentiated tumors, ES cell marker genes, such as *SOX2*, *OCT4*, and *c-MYC*, are preferentially aberrantly activated (36). Hepburn et al. (37) reported the characterization of ATP-binding cassette (ABC) superfamily G member 2 (ABCG2) in multiple BC cell lines and NMIBC samples along with the successful isolation of SP cells with high ABCG2 expression. Compared with non-SP cells with low ABCG2 expression, *SOX2* gene enrichment was increased in the SP cells, and stronger colony-forming ability was exhibited. Although there may exist a regulatory relationship between the expression of ABCG2 and *SOX2*, it is not elaborated in the text. In 2017, Zhu et al. (38) obtained *SOX2*+ cell subsets *via* flow cytometry and found that these subsets also expressed BCSC markers Keratin-14 and CD44v6. They also found that a subset of *SOX2*+ cells maintained BC progression, and the ablation of this subset led to tumor regression; thus, *SOX2* is also a marker for BCSCs (38). Most of the markers were identified based on the cell surface markers of SP, and it was confirmed that SP is enriched in BCSCs both *in vivo* and *in vitro* (39). It has been suggested that the aberrant activation of these pluripotent markers could be involved in therapeutic resistance mechanisms in carcinoma (40). Therefore, it is increasingly apparent that effective therapies are required to target the markers of BCSCs to address the issue of BC chemotherapy resistance. In this regard, *SOX2* is a potential target, and it has been implicated in the promotion of BC progression and chemoresistance.

## 4 Significance of SOX2 in bladder cancer

In normal human tissues, the role of transcription factor *SOX2* is to maintain the self-renewal of embryonic stem cells and generate induced pluripotent stem cells (41). The upregulation of *SOX2* has been detected in small-cell lung cancer and cancers of the prostate, colon, breast, and esophagus (42–46). In BC, the *SOX2* gene is abnormally activated. According to different stages and grades, the expression frequency of *SOX2* varies. The expression frequency of *SOX2* in MIBC and high-grade NMIBC is higher than in low-grade NMIBC (47).

In prognosis, Ruan et al. (48) revealed that in an NMIBC cohort, *SOX2* expression was significantly correlated with tumor size, number, and histological grade. Recurrence-free survival was higher in patients with low *SOX2* expression compared to those with high *SOX2* expression, and the difference was statistically significant. Multivariate cox regression analysis indicated that *SOX2* is an independent prognostic factor for recurrence-free survival in patients with stage-T1 BC. Based on immunohistochemical results in the MIBC patient cohort, Matias et al. (49) took a different view; cytosolic staining for *SOX2* was not associated with patient prognosis; differences in *SOX2* cytoplasmic expression were not associated with patient histological grade; and weak staining in the cytoplasm predicted a poorer prognosis, but this difference was not statistically significant.

With regard to treatment outcome, high *SOX2* expression was strongly associated with chemoresistance. A higher proportion of patients in the chemoresistant group had high *SOX2* expression compared to those in the pre-NAC chemo-sensitive group, and a higher proportion of patients in the post-NAC chemoresistant group had high *SOX2* expression compared to those in the pre-NAC chemoresistant group (49). However, the sample size of the MIBC cohort studied by Matias’ team was small and the differences were not statistically significant, so their results were not sufficiently convincing.

## 5 Regulation of *SOX2* expression in bladder cancer

SOX2 is an essential transcription factor, and due to the lack of a suitable binding site, it is almost impossible to directly regulate by inhibitors (38, 50). In most current studies, the expression of *SOX2* is indirectly regulated by modulating the expression of a certain protein or the conduction of a certain signaling pathway (28, 39, 51, 52). In this section, various mechanisms regulating *SOX2* expression are discussed.

### 5.1 Regulation by microRNA

In eukaryotes, not all RNAs are translated into proteins, and non-coding RNAs (ncRNAs) are a category of RNAs that do not encode proteins. Such RNAs are classified as small ncRNAs and long ncRNAs; small ncRNAs include ribosomal RNA, transfer RNA, and microRNA (miRNA) (53). Evidence suggests that lncRNAs and miRNAs drive the occurrence and progression of human carcinomas by mediating the transcriptional and post-transcriptional levels of genes, i.e. mRNA degradation and protein translation failure (54).

MicroRNA is about 21–23 nucleotides in length and regulates the proliferation, differentiation, and apoptosis of BC cells (55). In 2015, Tomomi (56) reported that the overexpression of miR-145 can inhibit the expression of syndecan-1 and induce abnormally high *SOX2* expression in BC cells, thereby inducing BC cell differentiation. Primarily, miRNAs modulate the post-transcriptional levels of downstream target genes that suppress protein translation by complementing the bases of the 3’ untranslated region (3’UTR) of the target gene’s mRNA (57). It was demonstrated that the expression of miR-200c, which was upregulated in BC, complemented the 3’UTR of SOX2 mRNA, thereby inhibiting *SOX2* transcription (58). In 2019, Wang et al. (59) reported that the rs2910164 single-nucleotide polymorphism in the miR-146a precursor regulated the expression of miR-146a. The target gene of miR-146a is COX2 mRNA, and COX2 regulated the expression of *SOX2*. The overexpression of miR-146a resulted in the downregulation of COX2 transcription, which in turn, downregulated the transcription and translation of the *SOX2* (**Table 1**).

**Table 1:** Summary of non-coding RNAs regulating SOX2 expression in bladder cancer.

Non-coding RNA	up/downregulationof SOX2	function	Expression withrespect to BC	Cell lineused	Ref.
miR-145	Up	Promote cell differentiation	Promotive	T24/KU7	(50)
miR-146a	Down	A biomarker for BC relapse	Suppressive	T24/RT4	(53)
let-7	Down	Inhibit cell survival And sphere formation	Suppressive	BFTC905/BFTC909	(54)
lnc-LBCS	Down	Inhibit self-renewal, chemoresistance, and tumor initiation of Bladder CSCs	Suppressive	UM-UC-3/5637	(55)
lncRNA AK023096	Up	Promote self-renewal of Bladder CSCs	Promotive	UM-UC-3/J82	(56)
SOX2OT	Up	Promote metastasis and the stemness phenotype of Bladder CSCs	Promotive	SW780/5637	(58)
miR200-c	Down	Inhibit sphere formation	Suppressive	SW780	(58)
circFLNA	Down	Inhibit cell proliferation, migration, invasion and EMT	Suppressive	5637	(60)
miR-216a-3p	Up	Promote cell viability, proliferation, invasion and migration	Promotive	5637	(60)

The COX2/PGE2 signaling pathway induces the methylation of the promoter of the let-7 gene and downregulates its expression. The high-mobility group AT-hook 2 (*HMGA2*) is the downstream target gene of let-7; HMGA2 binds to the promoter region of *SOX2* and upregulates its expression, while let-7 complements the HMGA2 mRNA 3’UTR to inhibit HMGA2 translation, which in turn, inhibits the transcription and translation of the *SOX2* gene (60) (**Table 1**).

### 5.2 Regulation by long non-coding RNA

**Figure 2:**
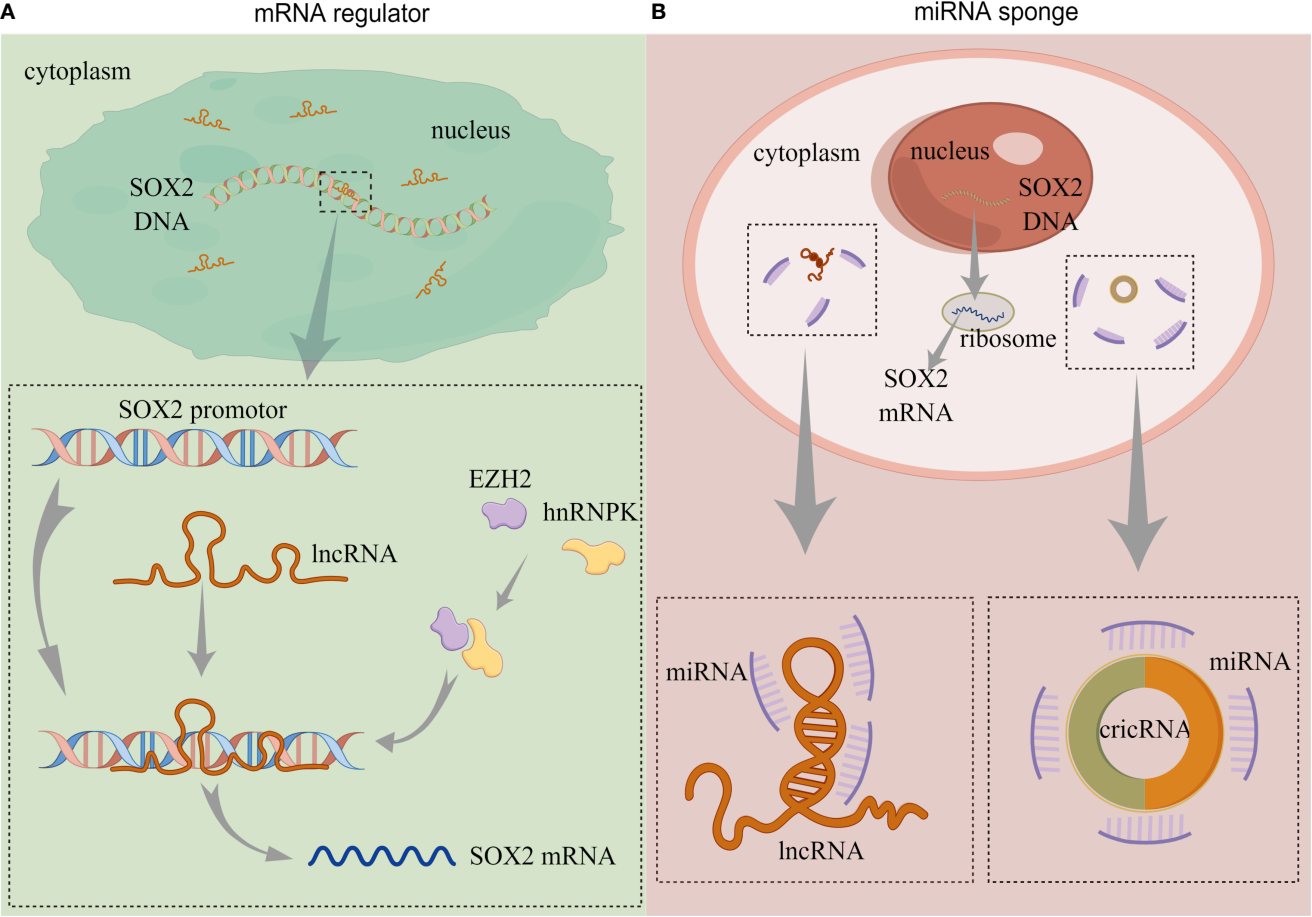
The functions of non-coding RNAs. **(A)** lncRNA directly regulates *SOX2* expression. **(B)** lncRNA and circRNA act as miRNA sponges to regulate *SOX2* expression. (lncRNA, long-non coding RNA; circRNA, circularRNA; miRNA, microRNA). By Figdraw.

In BC, several lncRNAs capable of regulating *SOX2* expression have been reported, and their modulation of such expression can be summarized in two approaches: 1) lncRNA directly regulate *SOX2* expression, and 2) lncRNA as miRNA sponges to regulate *SOX2* expression (**Figure 2**).

Chen et al. (61) reported that an lncRNA called Low was expressed in BCSCs (lnc-LBCS); it could regulate the expression of *SOX2*, bind to the *SOX2* promoter, and upregulate the level of H3K27me3. Furthermore, lnc-LBCS regulates the expression of heterogeneous nuclear ribonucleoprotein K (hnRNPK) and zeste homolog 2 (EZH2). Long non-coding LBCS mediates the recruitment of the hnRNPK-EZH2 complex to the *SOX2* promoter, inducing H3K27me3 levels and thereby repressing *SOX2* transcription and translation (61) (**Figure 2A**). A recent study confirmed this regulation, and the AK023096 lncRNA also promoted *SOX2* expression by recruiting hnRNPK to mediate the methylation of the *SOX2* promoter (62) (**Figure 2A**). It has been shown that lncRNA can function as miRNA sponge in cancer cells, attaching to miRNAs as a competing endogenous RNA (ceRNA), thus relieving the inhibitory effect of miRNAs on their target genes (63). In 2020, Zhan et al. (64) showed that *SOX2*-overlapping transcript (SOX2OT) is an lncRNA located in the intron region of *SOX2*, which is dominantly detected in the cytoplasm of BC cells. The expression of SOX2OT is aberrantly activated in BC and upregulates *SOX2* expression. The knockdown of SOX2OT expression upregulates *miR200-c* expression, and silencing the expression of *miR200-c* reverses the downregulation of *SOX2* (64) (**Figure 2B**). Consequently, SOX2OT upregulates *SOX2* expression by sponging *miR200-c* in a ceRNA manner and inhibiting *miR-200c* complementation with the *SOX2* mRNA 3’UTR (**Table 1**).

### 5.3 Regulation by circular RNA

Circular RNA (circRNA) contain abundant miRNA binding sites and can act as miRNA sponges to regulate the post-transcriptional levels of their target genes (65) (**Figure 2B**). Lin et al. (66) demonstrated that the expression of circRNA, which is circularized from exons 9–15 of the filament protein A (*FLNA*) gene (circFLNA), is downregulated in BC. The overexpression of circFLNA resulted in the significant downregulation of *SOX2* expression. The target gene of circFLNA was miR-216a-3p, and the overexpression of miR-216a-3p upregulated the expression of *SOX2*. Their studies also revealed that circFLNA served as miRNA sponge and competitively adsorbed miR-216a-3p, thereby upregulating B-cell translocation gene 2 (BTG2) expression (66) (**Table 1**). In their publication, *SOX2* was positively regulated by miR-216a-3p, but the specific mechanism was not elucidated. BTG2 was a downstream target of miR-216a-3p, and whether the expression of *SOX2* was regulated by BTG2 was not covered.

### 5.4 Regulation by epithelial–mesenchymal transition (EMT)

Investigations have revealed that EMT is involved in cancer invasion, metastasis, and drug resistance, and it is associated with poor prognosis in BC (67). Transforming growth factor β1 (TGF-β1), an EMT inducer, induced EMT in HTB-9 cells and upregulated the expression of *SOX2* (68). Romaila et al. (69) reported that *Escherichia coli* caused EMT induction in T24 cells and upregulated *SOX2* expression. Migita et al. demonstrated that TGF-β1 induced EMT in MIBC (i.e., UM-UC-3, T24) and NMIBC- (RT-4, JTC-30) derived cells and confirmed that in the latter, EMT significantly upregulated the expression of *SOX2* (70). Pan et al. (52) reported that the dissociation of the OCT4–SOX2 compound in BC contributed to specific differentiation signals induced by EMT. In addition, the knockdown of *SOX2* expression in 5637 cells inhibited EMT (71). In part, EMT promotes the expression of marker genes for CSCs, such as *SOX2* in BC cells, and in turn, the dissociation of *SOX2* promotes EMT in BC cells.

### 5.5 The effect of epigenetic modification on *SOX2*

#### 5.5.1 RNA modification

N6-methyladenosin (m^6^A) is the most prevalent modification in higher organism mRNAs. Modifications of m^6^A have an influential role in the induction of BC occurrence and invasiveness (72). It was suggested that methyltransferase-like 3 (METTL3) upregulates the m^6^A modification of AF4/FMR2 family member 4 (AFF4) mRNA and upregulates the expression of AFF4, which in turn binds directly to the *SOX2* DNA promoter region and regulates the transcription of *SOX2* (73). Both MYC and *SOX2* are major regulators of self-renewal and tumorigenicity in CSCs, and METTL3 directly regulates m^6^A modifications on MYC mRNA and upregulates MYC transcription (74). Therefore, METTL3 may mediate *SOX2* expression at both the transcriptional and post-transcriptional levels, potentially affecting BC genesis and invasion.

#### 5.5.2 Protein modification

ChlA-F, a conformational derivative of Cheliensisin A, promotes the degradation of the SOX2 protein rather than inhibiting the transcription of SOX2. Further studies found that ChlA-F treatment upregulated both the transcription and translation of ubiquitin specific peptidase 8 (USP8) and that the knockdown of USP8 reversed the downregulation of SOX2 resulting from ChlA-F treatment. Additionally, ChlA-F induced the expression of HuR, which binds to USP8 mRNA and increases its stability. Therefore, ChlA-F promotes the ubiquitination and protein degradation of SOX2 by inducing HuR expression, whereby it upregulates the expression of USP8 and acts as an E3 ligase (58). ChlA-F regulates not only SOX2 transcription but also the stability of the SOX2 protein.

## 6 Role of SOX2 in bladder cancer treatment resistance and potential treatment strategies

In recent years, although considerable improvements have been achieved in the treatment of BC, the mortality rate due to chemotherapy resistance has increased, again confirming that the development of novel targeted drugs is an effective treatment for BC. In this section, the role of *SOX2* in BC treatment is summarized, and potential therapeutic strategies that target *SOX2*-related signaling pathways are discussed.

### 6.1 Potential role of SOX2 in BC immunotherapy

Among intravesical instillations after TURBt, *Bacillus Calmette–Guérin* (BCG) is the most successful method to treat and prevent recurrence or progression of NMIBC (2, 8). Peng et al. showed that the *SOX2*-expressing CD133+ cell population was more resistant to BCG treatment compared to the CD133- J82 population (35). However, the correlation of this resistance effect with *SOX2* has not been reported. In addition, the complex comorbidities and side effects caused by BCG can severely affect patients’ quality of life and are not suitable for all patients (75). The approved application of immune checkpoint inhibitors alleviates this situation, with inhibition of programmed cell death protein-1/programmed cell death protein ligand 1 (PD-1/PD-L1) and cytotoxic T-cell antigen (CTLA4) being the main immune checkpoints (76). Recent study showed that *SOX2* expression might be associated with PD-L1 expression. BC cells co-cultured with macrophages form tumor-hybrid cells (THC) with immunomodulatory ability (77). *SOX2* expression was more abundant in THC compared to BC cells. Treatment of THC with phenylbutyrate, an immunomodulator and antitumor compound, downregulated *SOX2* expression and upregulated PD-L1 expression simultaneously (78). However, the exact mechanism involved is unclear, and no relevant references have reported an association between *SOX2* and response rates to immune checkpoint inhibitors.

### 6.2 Potential therapeutic strategies for targeting SOX2-related signaling pathways

#### 6.2.1 MAPK signaling pathway

**Figure 3:**
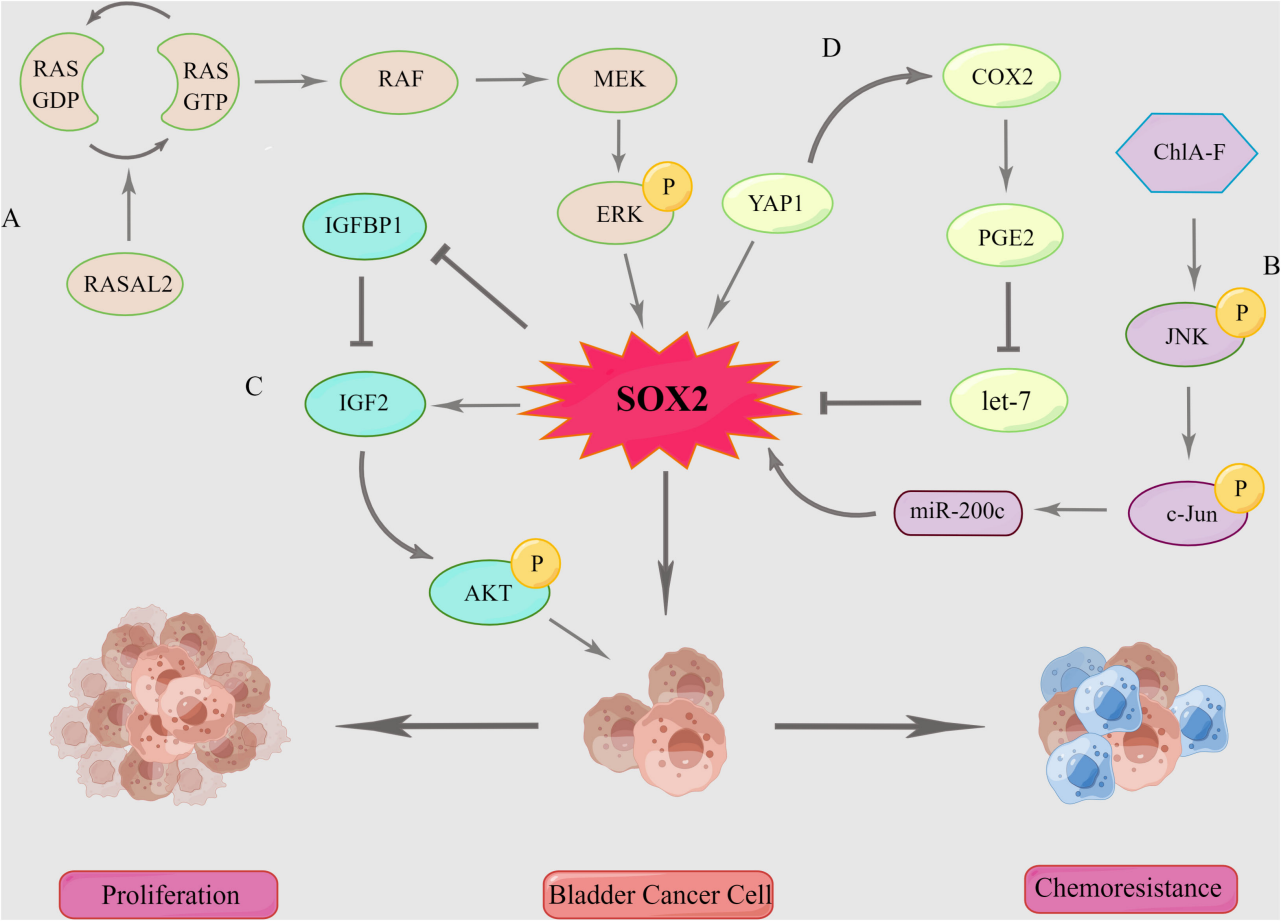
SOX2-related signaling pathway in bladder cancer. **(A)** MAPK/ERK signaling pathway. **(B)** MAPK/JNK signaling pathway. **(C)** SOX2-IGF2-AKT signaling pathway. **(D)** COX2/PGE2 Axis and YAP1-SOX2 signaling pathway. “P” stands for phosphorylation. By Figdraw.

Mitogen-activated protein kinase (MAPK) is widely expressed in multicellular organisms and has significant function in cell proliferation, differentiation, migration, and invasion (79). It can be classified into four subfamilies, namely, ERK, p38, JNK, and ERK5; each of these represents a pathway, with the ERK and JNK signaling pathways being the most deeply interconnected with BC (80). Hepburn et al. (37) reported that phosphorylated ERK (pERK) is aberrantly activated in SP cells and that MEK-specific inhibitors significantly suppress the colony-forming ability of such cells. Mitogen-activated protein kinase signaling pathways may be linked to the maintenance of marker genes in BCSCs. However, their study was limited by the mechanism. Hui et al.’s research (71) revealed the link between the MAPK/ERK signaling pathway and the expression of *SOX2*. RASAL2, a RAS GTPase-activating protein (RAS GAP), which is downregulated in BC tissues and cells and regulates the spheroid and colony-forming abilities of BC cells. It was reported that the knockdown of RASAL2 resulted in the upregulation of pERK expression, while the overexpression of RASAL2 resulted in its downregulation. Their studies also showed that MEK-specific inhibitors significantly downregulated *SOX2* expression in BC cells and inhibited spheroid and colony-forming abilities (71) (**Figure 3A**). Therefore, RASAL2 inhibits *SOX2* expression *via* the MAPK/ERK signaling pathway, which in turn, inhibits BC cell migration and stemness.

ChlA-F selectively induces JNK and c-Jun phosphorylation and upregulates *miR200-c* transcription, which inhibits SOX2 protein translation and suppresses BC cell invasion (58) (**Figure 3B**). Therefore, targeting the ERK and JNK signaling pathways to inhibit the expression of *SOX2* may be a potential target for BC therapy.

#### 6.2.2 AKT-related signaling pathway

Tannic acid (TA) significantly inhibited BC cell viability and suppressed *SOX2* expression (81). Further studies revealed that the treatment of cells with TA inhibited the AKT phosphorylation (pAKT) of Ser473 (81). Denise et al. (82) reported inducible nitric oxide synthase (iNOS) to be associated with the aggressiveness and recurrence of BC. The inhibition of iNOS expression significantly downregulated *SOX2* expression and reduced matrix metallopeptidase 2 (MMP-2) activity in a mouse BC model (83). Evidence confirms that *SOX2* induces both MMP-2 activity and the PI3K/AKT pathway involved in laryngeal cancer invasion (84). Thus, iNOS may regulate BC recurrence by upregulating *SOX2* expression and inducing both MMP-2 activity and AKT phosphorylation.

Chiu et al. (85) clarified the underlying mechanism of *SOX2* regulation of pAKT. In a low-serum culture, *SOX2* maintained the viability of BC cells, and pAKT levels were upregulated in *SOX2*-expressing BC cells. *IGF2* is a downstream gene of *SOX2*, which upregulates H3K4me3 in the *IGF2* promoter. Thus, *SOX2* upregulates pAKT of ser473 by inducing the expression of *IGF2* and suppressing the expression of *IGFBP1*, thereby promoting the proliferation and invasion of BC cells (85) (**Figure 3C**). However, Wang et al. (86) reported that in esophageal cancer, pAKT upregulates *SOX2* phosphorylation (pSOX2) at T116 and protects the SOX2 protein from ubiquitination degradation by Ubiquitin protein ligase E3 component n-recognin 5 (UBR5). It remains unknown if pAKT directly regulates the expression of *SOX2* in BC, and the mechanism between AKT and *SOX2* requires further investigation.

#### 6.2.3 COX2/PGE2–SOX2 axis and YAP1-SOX2 signaling pathway

Arsenic is a widespread environmental contaminant and an essential factor in the pathogenesis of BC (87). In a long-term arsenic-induced (AS) *in vitro* model of the human normal urothelial cell line HUC1, the expression of *SOX2* was upregulated in AS cells, and it was an integral and key factor for AS stem cell properties. Furthermore, the COX2 inhibitor was found to downregulate *SOX2* expression, while PGE2 upregulated *SOX2* expression. Therefore, *SOX2* expression is regulated by COX2/PGE2 (88).

Due to the aberrant activation of the epidermal growth factor receptor (EGFR) pathway in BC, EGFR has been shown to be a potential target for the basal subtype of MIBC (89). The use of EGFR-targeted treatment resulted in the upregulation of both PGE2 and SOX2, which may be attributed to the enrichment of CSCs. These results indicate that the combined application of EGFR and COX2 inhibitors to AS cells resulted in the significant, but not complete, suppression of *SOX2* expression compared with treatment with EGFR inhibitors alone (88). This is explained in a separate report by Ooki et al. (60), with Yes-associated protein 1 (YAP1) binding to the *SOX2* enhancer region and upregulating *SOX2* expression. The *COX2* is the downstream target gene of YAP1, but YAP1 regulates *SOX2* expression not exclusively *via* the COX2/PGE2 axis (88, 90). The YAP1–SOX2 and COX2/PGE2–SOX2 signaling pathways are modulated independently, although there is a negative feedback mechanism between the two (60) (**Figure 3D**). Additionally, EGFR upregulates YAP1 expression *via* the activation of the PI3K/AKT signaling pathway (91). Accordingly, due to the negative feedback regulation of COX2 inhibition, the combined inhibition of EGFR and COX2 is not accompanied by the upregulation of YAP1 expression (60). So, the strategy of co-inhibition boosted the short-term anti-tumor effect if the interaction between molecules and the potential for additional side effects is considered.

#### 6.2.4 Other classical signaling pathways

The primary roles of the Wnt/β-Catenin signaling pathway are the mediation of cell proliferation, metastasis, and differentiation (92). The Wnt/β-Catenin signaling pathway is aberrantly activated in BC and is associated with maintaining BC resistance to gemcitabine and paclitaxel (93). Guerrero et al. (94) reported *SOX2* expression to be abnormally upregulated in the resistant HT1197 cell line compared with the 5637 cell line, which was not resistant to paclitaxel.

The aberrant activation of the hedgehog signaling pathway may be relevant to chemoresistance in BC (95). The expression of *SOX2* was higher in T24 with cisplatin resistance than in WT T24 cells, inhibiting the expression of major hedgehog pathway markers; furthermore, *SOX2* expression was also inhibited (96). Sonic hedgehog (Shh) is the main signaling protein of the hedgehog pathway, and BCSCs may originate from the basal urothelium expressing Shh (97). The Shh signaling pathway regulates BC cell EMT, which in turn, upregulates *SOX2* expression (68). Although *SOX2* is regulated by these classical pathways in BC cell lines with resistance, these reports do not explore the detailed mechanisms by which *SOX2* governs BC chemoresistance. Accordingly, the study of genes downstream of *SOX2* may be a future research direction.

## 7 Conclusion and future perspectives

Many studies have linked *SOX2* with human carcinomas. The aberrantly high expression of *SOX2* is associated with chemotherapy resistance in glioma, prostate cancer, rectal cancer, and other cancers. After the downregulation of *SOX2* expression, tumors can recover their sensitivity to drugs (98). This review focused on the role of *SOX2* in BC, with multiple reports describing the abnormally high expression of *SOX2* in BC tissues, which is correlated with clinical grade and prognosis and is an independent prognostic factor for BC. Due to the special identity of *SOX2* transcription factors, it is almost impossible to directly inhibit the expression of *SOX2 via* inhibitors. Numerous studies have reported that ncRNA, EMT, epigenetics, and signaling pathways mediate BC progression and chemoresistance by modulating the transcription or translation of *SOX2*, which has tremendous potential as a target for BC therapy. Notably, the effect mechanism of chemotherapeutic agents, such as cisplatin, is mainly *via* intracellular DNA structure, where it induces apoptosis. Under selective pressure, chemoresistance leads to an enrichment of CSCs in tumors, to some extent reinforcing drug resistance. As reported in the literature, the co-inhibition of multiple sites may be a new direction for the targeted modulation of *SOX2*, but it may cause additional side effects. While numerous studies on the regulation of *SOX2* have been reported, only a few have examined the genes downstream of *SOX2*. Moreover, adult stem cells exist in various tissues of the human body and also express *SOX2*. Therefore, how to target and regulate the abnormally high expression of *SOX2* in BC without affecting the homeostasis of normal tissue stem cells may also be a crucial point to be addressed.

In summary, in-depth research into the mechanisms of BC drug resistance and the identification of suitable targets and regulatory pathways to improve the responsiveness are essential future research directions for improving the current status of BC therapeutics. Despite reports of several mechanisms and signaling pathways that mediate *SOX2*, none have been used in clinical trials. Thus, there is still a long way to go in the field of BC targeted therapy. Complicated interactions between signaling pathways, protein-signaling pathways, and protein–protein interactions pose numerous obstacles. Exploring the critical pathways that regulate drug resistance in BC and developing precisely targeted drugs for clinical trials are needed for the further development of targeted therapies.

## Author contributions

GC searched for literature and wrote the first draft of this article. YC and GC edited tables and figures. RX, XZ and GW reviewed the manuscript and polished the grammar. All authors contributed to the article and approved the submitted version.

## Funding

This work was supported by the National Natural Science Foundation of China (No. 81860456); Key Project of Key Research and Development Plan of Jiangxi Province (Grant No. 20212BBG71013).

## Conflict of interest

The authors declare that the research was conducted in the absence of any commercial or financial relationships that could be construed as a potential conflict of interest.

## Publisher’s note

All claims expressed in this article are solely those of the authors and do not necessarily represent those of their affiliated organizations, or those of the publisher, the editors and the reviewers. Any product that may be evaluated in this article, or claim that may be made by its manufacturer, is not guaranteed or endorsed by the publisher.
